# Characterizing the complete chloroplast genome of the *Impatiens davidii* (Balsaminaceae)

**DOI:** 10.1080/23802359.2022.2048211

**Published:** 2022-03-09

**Authors:** Chenhua Fu, Xiyan Chen, Tongjian Li, Anpei Zhou

**Affiliations:** a School of Pharmacy and Life Science, Jiujiang University, Jiujiang, China; b Jiujiang Senior Technical Schools, Jiujiang, China; c Research Center for Jiangxi Oil-tea Camellia, Jiujiang University, Jiujiang, China

**Keywords:** *Impatiens davidii*, ornamental flower, chloroplast genome

## Abstract

*Impatiens davidii* Franch, 1886 is a rare ornamental flower used in gardens and has high economic value. In this study, we characterized the chloroplast genome of *I. davidii* and analyzed its phylogenetic relationship with other *Impatiens* species. The length of the complete chloroplast genome sequence of *I. davidii* is 152,214 bp, with a GC content of 36.9%. The chloroplast genome shows a typical quadripartite structure with a pair of inverted repeats (IRs) of 25,634 bp, separated by one large single copy (LSC) region of 83,128 bp and one small single copy (SSC) region of 17,818 bp. We annotated 125 genes, of which there were 85 protein-coding genes, 32 tRNA genes, and 8 rRNA genes. The Bayesian phylogenetic tree strongly supports that *I. davidii* has a close phylogenetic relationship with a group including *I. piufanensis* and *I. alpicola*.

*Impatiens* plants, rare ornamental flowers in gardens, contain more than 1000 species and are distributed over the whole Northern Hemisphere and tropical zone (Vrchotová et al. 2011). China has rich *Impatiens* germplasm resources with over 220 species, a few of which play important roles in gardens (Cheng 1999). *Impatiens davidii* Franch, 1886 is an endemic species that mainly occurs in the Lushan Mountain and its adjoining areas, and its study is helpful for genetic improvement and variety breeding of *Impatiens* flowers (Cheng 1999). Chloroplast genomes are characterized by highly conserved sequences and structures because of their nonrecombinant, haploid and uniparentally inherited nature (Birky 2001; Wicke et al. 2011). Achieving good chloroplast genomic information is helpful to understand genomic variations and contributes to further physiological molecular and phylogenetic studies (Zhong et al. 2019). Here, we first report the chloroplast genomic information of *I. davidii* and analyze its phylogenetic location in the genus *Impatiens*. The annotated genomic sequence has been submitted to GenBank under the accession number MZ424444.

Samples of *I. davidii* were collected from Lushan Mountain (29°35′40.21ʺN,115°59′9.6ʺE) and the total genomic DNA from the fresh leaves was extracted with the DNAprep Pure Plant Kit (Tiangen Biotech, Beijing, China). An *I. davidii* specimen was deposited in the Laboratory of Molecular Biology of Jiujiang University (Anpei Zhou, Email: 6090078@jju.edu.cn) under the voucher number ID-AP1. Total DNA was used to generate libraries on an Illumina NovaSeq 6000, and approximately 3 Gb raw data were produced with 150 bp paired-end read lengths. GetOrganelle software (Jin et al. 2020) was used to assemble the complete chloroplast genome of *I. davidii*. The obtained scaffolds were adjusted to produce chloroplast genome sequences using Bandage software (Wick et al. 2015) and the initial annotation was completed using Geneious R8 software (Biomatters Ltd, Auckland, New Zealand). The codons were checked and adjusted by comparison with the reference genome *I. pritzelii*.

The chloroplast genome of *I. davidii* is a typical circular double stranded DNA molecule. The genome length is 152,214 bp with a GC content of 36.9%, containing a large single copy (LSC) region of 83,128 bp, a small single copy (SSC) region of 17,818 bp, and a pair of inverted repeats (IRs) of 25,634 bp. The chloroplast genome of *I. davidii* encodes 125 functional genes, including 85 protein-coding genes, 32 tRNAs, and 8 rRNAs. Among the genes, eleven genes (*atpF*, *ndhA*, *ndhB*, *rpl2*, *rpoC1*, *rps12*, *rps16*, *petB*, *trnI*, *trnA-UGC*, *tRNA-Ala*) have one intron and two genes (*ycf3*, *clpP*) have two introns. The number of chloroplast genes is different between *I. davidii* and the reference *I. pritzelii*. Four genes (*pbf1*, *tRNA-Ala*, *trnI*, *trnM-CAU*) are found to be lost in the reference *I. pritzelii*, and eight genes (*psbN*, *trnfM-CAU*, *trnI-CAU*, *trnI-GAU*, *trnK-UUU*, *trnL-UAA*, *trnP-GGG*, *trnV-UAC*) are absent in *I. davidii*. Mohanta et al. (2020) found that six chloroplast genes (*chlB*, *chlL*, *chlN*, *psaM*, *psb30*, *rpl21*) were lost in the angiosperm or magnoliid lineage, which is supported by our results.

The phylogenetic location of *I. davidii* in the genus *Impatiens* was examined with fully sequenced chloroplast genome. Six chloroplast genome sequences of five *Impatiens* species (*I. alpicola, I. glandulifera, I. hawkeri, I. piufanensis,* and *I. pritzelii*) were obtained from GenBank, and one *Hydrocera triflora* species in the Balsaminaceae family was used as the outgroup. A total of eight complete chloroplast sequences were aligned through MAFFT v7 software (Katoh and Standley 2013). Based on the Akaike information criterion (AIC) derived from ModelTest 3.7 software (Posada and Crandall 1998), the best-fitting nucleotide substitution model was GTR + I + G. Bayesian inference was employed to reconstruct a phylogenetic tree using MrBayes software (Ronquist and Huelsenbeck 2003). In this step, the parameter settings were 1,000,000 generations, and the posterior probability was estimated using the Markov chain monte carlo (MCMC) method. According to the results, *I. davidii* can be considered sister to a group including *I. piufanensis* and *I. alpicola* (Figure 1).

**Figure 1.**
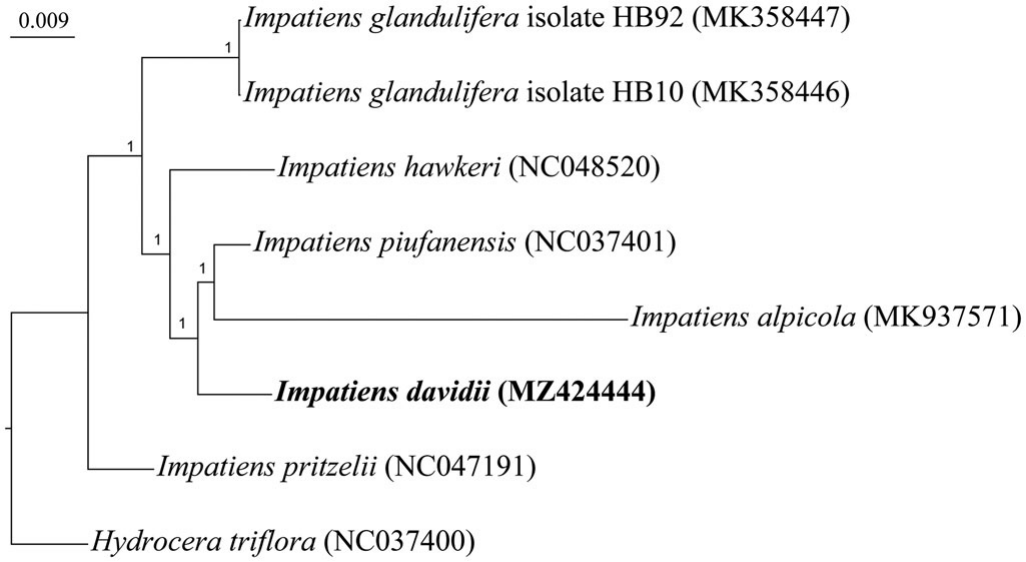
Bayesian phylogenetic tree based on the complete chloroplast genome sequences. Six chloroplast genome sequences of five *Impatiens* species are downloaded from GenBank and *Hydrocera triflora* is set as the outgroup. The phylogenic tree is constructed by the Bayesian inference method with 1,000,000 generations. The posterior probability values are shown at nodes.

## Data Availability

The genome sequence data that support the findings of this study are openly available in GenBank of NCBI under the accession number MZ424444. The associated BioProject, SRA, and Bio-Sample numbers are PRJNA766029, SRR16043999, and SAMN21600381, respectively.
